# Full‐Thickness Corneal Perforation Secondary to Monopolar Cautery Burn During Cosmetic Blepharoplasty in a Young Adult: Successful Management With Cyanoacrylate Glue

**DOI:** 10.1002/ccr3.72071

**Published:** 2026-02-23

**Authors:** Rahim Saffari, Mojtaba Abrishami, Seyed Ali Ebrahimi, Amin Maleki

**Affiliations:** ^1^ Eye Research Center Mashhad University of Medical Sciences Iran

**Keywords:** blepharoplasty complication, corneal burn, corneal perforation, cyanoacrylate glue, monopolar cautery

## Abstract

Thermal corneal injuries are uncommon but potentially vision‐threatening. Full‐thickness corneal perforation caused by monopolar electrocautery during cosmetic blepharoplasty is exceptionally rare and scarcely reported in the literature. We report a 27‐year‐old healthy man who sustained an accidental central full‐thickness corneal perforation during elective upper eyelid blepharoplasty due to inadvertent contact with monopolar cautery. The patient presented with severe ocular pain, markedly reduced vision, and a shallow anterior chamber. Prompt intervention was performed under sterile conditions. The corneal perforation was sealed using N‐butyl cyanoacrylate tissue adhesive, followed by placement of a bandage contact lens. Adjunctive topical and systemic medications were administered to prevent infection, control inflammation, and inhibit collagenolysis. At one‐week follow‐up, the adhesive remained intact with a stable anterior chamber and no evidence of leakage or infection. Visual acuity improved to 20/200, limited by central stromal opacity. Although long‐term follow‐up was unavailable, immediate tectonic stabilization was successfully achieved. This case highlights a rare but severe ocular complication of cosmetic blepharoplasty. Early recognition and rapid application of cyanoacrylate glue can effectively preserve globe integrity in small corneal perforations. Proper ocular protection is essential during periocular surgical procedures to prevent such catastrophic injuries.

## Key Clinical Message

Full‐thickness corneal perforation secondary to monopolar cautery burn during blepharoplasty is an exceptionally rare but vision‐threatening complication. Immediate recognition and rapid sealing with cyanoacrylate glue can preserve globe integrity, prevent infection, and provide time for definitive visual rehabilitation. Proper intraoperative ocular protection is essential.

## 1 Introduction

Thermal burns of the cornea are uncommon but potentially sight‐threatening injuries. They typically result from direct exposure to high‐temperature sources such as molten metal, hot liquids, or surgical energy devices. Unlike chemical burns, which often produce diffuse and progressive tissue damage, thermal burns generally induce localized necrosis with sharply demarcated borders [1]. The severity of injury depends on the source temperature, exposure duration, and the area of the corneal surface involved [2].

Histopathologically, thermal energy induces coagulative necrosis of the corneal epithelium and stroma. Collagen fibrils undergo denaturation and contraction, leading to stromal opacity, thinning, or, in severe cases, immediate full‐thickness perforation [3]. Secondary inflammatory responses, including keratocyte apoptosis and enzymatic collagen degradation, may exacerbate stromal melting after the initial insult [4].

Clinically, corneal thermal burns range from superficial epithelial defects and stromal haze to full‐thickness perforations with aqueous leakage [5]. While superficial injuries often heal without sequelae, deep burns can cause scarring, secondary infection, and permanent visual loss. Unlike chemical burns, where limbal ischemia is a primary prognostic factor, outcomes in thermal burns are primarily dictated by injury depth [6].

Intraoperative thermal burns are rare but have been reported during phacoemulsification, diode laser procedures, and ocular surface surgery [7, 8]. Monopolar cautery, commonly used for hemostasis during blepharoplasty, can inadvertently injure the cornea if protective measures (metallic shields or lubricants) are absent or displaced. While ocular complications of blepharoplasty are generally minor, such as exposure keratopathy or superficial epithelial defects, full‐thickness thermal corneal injury is exceedingly uncommon [9].

Management of corneal perforations, whether traumatic, infectious, or iatrogenic, aims to restore globe integrity, prevent infection, and preserve vision. For small perforations (< 3 mm), cyanoacrylate glue is the standard temporizing intervention [10, 11]. It polymerizes rapidly on the corneal surface, providing a mechanical barrier that stabilizes the globe, reduces aqueous leakage, and allows time for definitive surgical intervention, such as keratoplasty. Additionally, cyanoacrylate exhibits bacteriostatic properties and inhibits collagenolysis, making it particularly suitable for acute stromal necrosis [12].

Herein, we report a young patient who developed a central full‐thickness corneal thermal defect during cosmetic blepharoplasty using monopolar electrocautery, which was successfully managed with cyanoacrylate glue.

## 2 Case Presentation

A 27‐year‐old otherwise healthy male underwent elective upper eyelid blepharoplasty at a private clinic. During intraoperative hemostasis, the monopolar cautery probe accidentally contacted the cornea, causing sudden severe ocular pain, and the procedure was immediately discontinued. The patient was urgently referred to our tertiary eye hospital approximately 2 h after the injury.

### 2.1 Examination Findings

**FIGURE 1:**
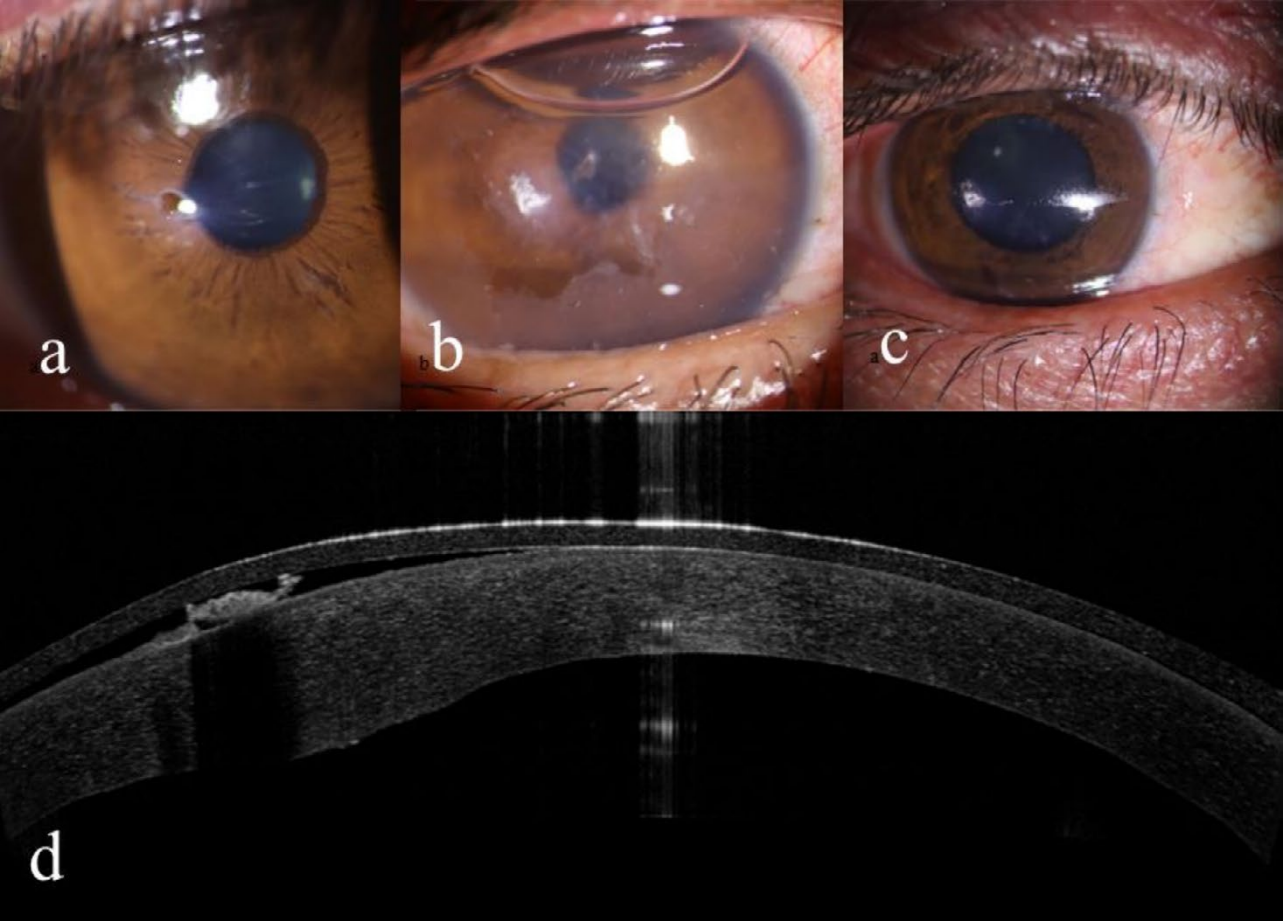
Slit‐lamp photographs before intervention demonstrate central full‐thickness corneal perforation with sharply demarcated whitish necrotic margins and stromal edema (a). Slit‐lamp photograph 2 days (b) and 1 week (c) after cyanoacrylate glue application, showing intact adhesive patch and stable anterior chamber. Corneal OCT image after glue application, demonstrating the central full‐thickness defects sealed with cyanoacrylate (d).

On presentation, best‐corrected visual acuity (BCVA) in the right eye was counting fingers at 1 m. Slit‐lamp examination revealed a 1 mm central full‐thickness corneal perforation with sharply demarcated whitish necrotic margins, overlying epithelial loss, and surrounding stromal edema, consistent with a thermal burn. No cautery residue or foreign material was observed on the corneal surface upon slit‐lamp examination. The anterior chamber was shallow and poorly formed; however, no iris prolapse, lens involvement, or fibrin reaction was observed. Intraocular pressure was not assessed at the initial visit due to the presence of an open globe. The fellow eye was unremarkable (Figure 1a).

### 2.2 Management

In the operating room, after prep and drape, under general anesthesia and sterile conditions, the ocular surface was copiously irrigated with balanced salt solution. Following a corneal stab incision, the anterior chamber was reformed with air, and the corneal surface was carefully dried. A small droplet of N‐butyl cyanoacrylate was then applied to seal the perforation, and a bandage contact lens was placed to protect the adhesive and enhance patient comfort. After the procedure, the anterior chamber depth was normalized, and Seidel's test was negative, confirming successful closure of the leak.

Postoperatively, the patient was treated with topical levofloxacin 0.5% six times daily, homatropine 2% twice daily, and frequent preservative‐free lubricants. In addition, oral doxycycline 100 mg twice daily and oral vitamin C 500 mg daily were prescribed to support corneal wound healing and reduce collagenolysis.

### 2.3 Outcome

At the 1‐week follow‐up, the cyanoacrylate glue remained intact, the anterior chamber was stable, and no leakage or signs of infection were detected. Re‐epithelialization was observed around the margins of the adhesive, and BCVA improved to 20/200, limited by central stromal opacity (Figure 1b,c). Corneal optical coherence tomography performed 2 days after glue application confirmed a central full‐thickness corneal defect successfully sealed with cyanoacrylate, associated with stromal thinning and a well‐formed anterior chamber (Figure 1d).

Unfortunately, follow‐up beyond 1 week was not possible because the patient was a foreign national and returned to his home country. Although long‐term outcomes could not be assessed, the immediate anatomical stabilization highlights the effectiveness of cyanoacrylate glue as an emergency temporizing measure in acute corneal perforations.

## 3 Discussion

Thermal corneal burns are uncommon but potentially sight‐threatening injuries. Unlike chemical burns, which often cause diffuse and progressive tissue damage, thermal injuries are typically more localized and may result in immediate collagen contraction and necrosis, occasionally leading to full‐thickness perforation [1, 3]. Intraoperative thermal injuries have been most frequently described during phacoemulsification (“wound burns”) and laser procedures [7, 8], whereas corneal thermal injury during cosmetic blepharoplasty is exceedingly rare.

In the present case, monopolar electrocautery used for intraoperative hemostasis likely came into direct or near‐direct contact with an unprotected cornea, resulting in a central full‐thickness thermal defect. Such cases are scarcely documented in the literature [9]. In an ex vivo porcine model comparing monopolar and bipolar devices, bipolar instruments produced less extensive tissue damage and reduced thermal spread compared with monopolar instruments, suggesting that bipolar devices may be inherently safer with respect to collateral thermal injury [13]. This underscores the critical importance of routine preventive measures during eyelid surgery, including the use of metallic eye shields and adequate corneal lubrication.

Management of corneal perforations aims to restore globe integrity, prevent infection, and preserve vision. Cyanoacrylate glue remains an effective temporizing measure for small corneal perforations, with optimal outcomes reported for defects ≤ 2 mm and acceptable results for perforations up to 3 mm in selected cases [10, 11]. The adhesive rapidly polymerizes on the corneal surface, forming a watertight seal that stabilizes the globe, reduces the risk of endophthalmitis, and provides temporary tectonic support until definitive surgical intervention, such as keratoplasty, can be undertaken. Additionally, cyanoacrylate exhibits bacteriostatic properties and inhibits collagenolysis, making it particularly advantageous in acute cases associated with stromal necrosis [12].

Reported success rates of cyanoacrylate glue in maintaining globe integrity during the early follow‐up period range from approximately 53% to 86% [11, 12]. Failure is more commonly observed in larger perforations, cases with progressive stromal melting, or when premature dislodgement of the adhesive occurs, necessitating repeat application or surgical repair. Potential complications include corneal neovascularization, inflammation, papillary conjunctivitis, and less commonly, elevated intraocular pressure [11]. Despite these limitations, cyanoacrylate glue remains a safe and effective first‐line option in emergency settings.

In the present case, immediate application of N‐butyl cyanoacrylate successfully sealed the perforation, restored anterior chamber integrity, and facilitated epithelial healing. Follow‐up was limited to 1 week because the patient returned to his home country; however, the rapid anatomical stabilization achieved highlights the effectiveness of cyanoacrylate glue as an emergency temporizing measure in acute full‐thickness corneal perforations.

This case illustrates a rare but potentially catastrophic complication of cosmetic blepharoplasty. Prompt recognition of corneal injury and immediate intervention were crucial in preserving globe integrity and preventing secondary infection. From a preventive standpoint, proper intraoperative ocular protection, including metallic eye shields and appropriate lubrication, is essential during periocular procedures to minimize the risk of thermal injury.

## 4 Conclusion

Full‐thickness corneal burns from monopolar cautery during cosmetic blepharoplasty are exceedingly rare but vision‐threatening. Prompt recognition and immediate sealing with cyanoacrylate glue can preserve globe integrity, prevent endophthalmitis, and provide time for definitive visual rehabilitation. This case highlights the critical importance of proper intraoperative ocular protection, including metallic eye shields and adequate lubrication, during eyelid surgery.

## Author Contributions

**Rahim Saffari:** conceptualization, project administration. **Mojtaba Abrishami:** supervision, writing – original draft. **Seyed Ali Ebrahimi:** data curation. **Amin Maleki:** writing – review and editing.

## Funding

The authors received no funding. The government or academic institutions do not fund the authors' work.

## Ethics Statement

Consent for publication: Written and comprehensive informed consent was obtained from the patient and he agreed to publish his data anonymously.

Ethics approval and consent to participate: The patient was completely informed about the procedure, and the institutional ethics committee approved the publication of the report.

## Conflicts of Interest

The authors declare no conflicts of interest.

## Data Availability

The data sets used and/or analyzed during the current report are available from the corresponding author upon reasonable request.
